# Next generation preventive neurology: how artificial intelligence and machine learning are reshaping Alzheimer’s disease research

**DOI:** 10.1186/s12993-026-00329-x

**Published:** 2026-03-31

**Authors:** Shivani Singh, Yashasvi Sharma, Prajjval Bhardwaj, Divyanshi Kothari, Anjali Chhikara, Vrinda Gupta, Dinesh Kumar, Neeraj Choudhary, Suresh Babu Kondaveeti

**Affiliations:** 1 School of Pharmaceutical Sciences, Jaipur National University, Jagatpura, Jaipur, Rajasthan India; 2 MM college of Pharmacy, Maharishi Markandeshwar deemed to be University, Mullana, Ambala, Haryana India; 3 Faculty of Pharmaceutical Sciences, ICFAI University, Baddi, Himachal Pradesh India; 4 Department of Pharmaceutics, GNA School of Pharmacy, GNA University, Phagwara, Punjab India; 5 Department of Pharmacognosy, GNA School of Pharmacy, GNA University, Phagwara, Punjab India; 6 Symbiosis Medical College for Women & Symbiosis University Hospital and Research Centre, Symbiosis International (Deemed University), Pune, 412115 India

**Keywords:** Neurology, Machine learning, Alzheimer's, AI, Convolutional neural networks

## Abstract

A neurological condition that worsens over time, Alzheimer’s disease (AD) is typified by memory loss, cognitive decline, and functional degradation. Traditional diagnostic techniques such as neuroimaging, cerebrospinal fluid biomarkers, and neuropsychological testing are often intrusive, costly, or insensitive in the early stages. Recent years have seen the emergence of AI and ML as game-changing technologies for AD risk assessment, early detection, and customized prevention. Using sophisticated models such as deep learning, convolutional neural networks (CNNs), and graph-based algorithms, AI-driven methods achieve high performance: CNNs, for example, have reached diagnostic accuracies of 94–99% for early AD and mild cognitive impairment using multimodal MRI and PET data. However, most reported performance metrics are derived from retrospective analyses and internal validation cohorts, with limited external validation across diverse populations. These methods include multimodal data integration from neuroimaging, genetics, and clinical records. Years before symptoms appear, AI-based frameworks can predict disease progression, identify modifiable risk factors, and guide individualized treatment plans. Future developments in federated learning and explainable AI (XAI) are promising, although data privacy, algorithmic bias, and ethical ramifications are concerns. Overall, AI and ML have a great deal of promise to transform the prevention of AD, enabling precision therapy and enhancing the lives of those who are at risk.

**Graphical Abstract:**

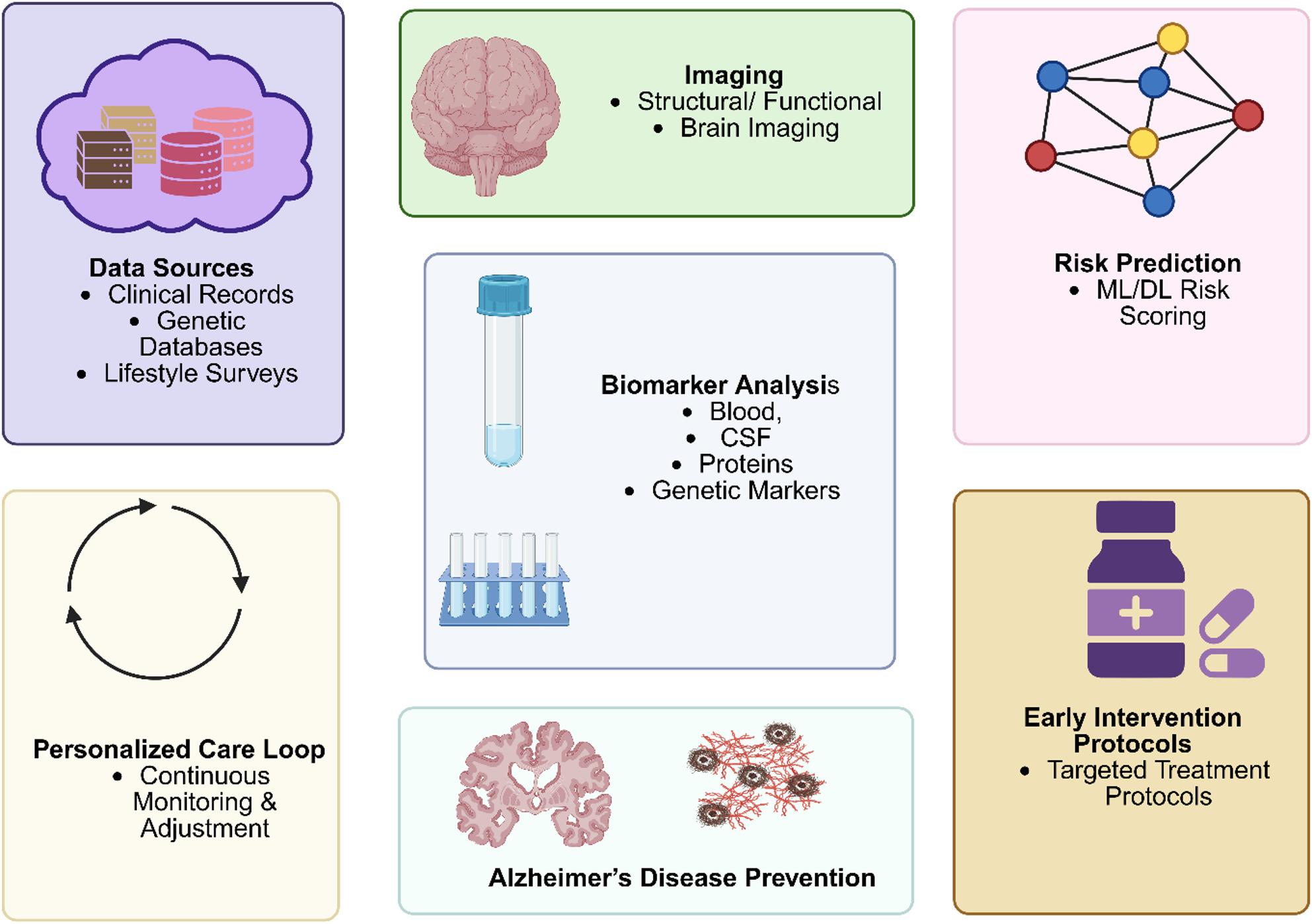

## Introduction

### Overview of Alzheimer’s disease

#### History

Alzheimer’s disease (AD) was first described in 1907 by Alois Alzheimer in a 51-year-old patient exhibiting memory loss and behavioural abnormalities, with postmortem evidence of senile plaques and neurofibrillary tangles. Subsequent studies by Oskar Fischer and Emilio Redlich further characterised these pathological features, while Emil Kraepelin later established AD as a distinct clinical entity. These early observations laid the foundation for modern neuropathological and clinical research on dementia Bermejo-Pareja and Ser, [14].

#### Prevalence and epidemiology

Alzheimer’s disease is a leading cause of dementia worldwide, affecting approximately 24 million individuals, with prevalence expected to increase substantially due to global population aging. According to NIA-AA guidelines, AD progresses through preclinical, mild cognitive impairment, and dementia stage. In the United States, approximately 6.9–7.2 million adults aged 65 years and older are affected, with prevalence rising markedly with age and disproportionately impacting Black and Hispanic populations. These epidemiological trends highlight the urgent need for scalable early detection and prevention strategies (Alzheimer’s Association, 2025).

#### Pathophysiology

The buildup of amyloid extracellular plaques and intracellular neurofibrillary tangles made mostly of tau are the main historical neuropathologic features [109]. In contrast to dementia, which is defined by increasing cognitive decline and limitations to functioning and is eventually fatal, memory, language, and problem-solving deficits may first manifest as moderate cognitive impairment (MCI), which is not severe enough to hinder functioning [54].

##### Amyloid metabolism in Alzheimer’s disease

In Alzheimer’s disease, abnormal processing of amyloid precursor protein leads to the accumulation of amyloid-β peptides, forming extracellular plaques that disrupt synaptic signaling and promote neuroinflammation. These pathological changes contribute to early neuronal dysfunction and cognitive decline Heneka et al., [39].

From an artificial intelligence perspective, amyloid deposition represents a quantifiable biomarker that can be extracted from PET and MRI imaging. Deep learning and ensemble models have been applied to estimate amyloid burden using standardized uptake value ratios, enabling early risk stratification and large-scale screening in preclinical populations. Such AI-based quantification supports preventive strategies by identifying high-risk individuals before symptom onset Maddury and Desai, [65].

This review primarily focuses on artificial intelligence–based approaches for early detection and risk stratification of Alzheimer’s disease, with particular emphasis on their role in guiding preventive interventions. While diagnostic, prognostic, and therapeutic applications are discussed, the central objective is to critically evaluate how AI can support preclinical identification and individualized risk assessment.

##### Tau accumulation in Alzheimer’s disease

Hyperphosphorylation of tau protein leads to the formation of neurofibrillary tangles, impairing axonal transport and correlating strongly with cognitive deterioration. Tau pathology is closely associated with disease progression and symptom severity Wei et al., [102].

AI-driven analysis of multimodal and multi-omics datasets has enabled the modeling of tau-related neurodegeneration using imaging, blood biomarkers, and genomic profiles. Machine learning models can predict longitudinal tau accumulation and disease trajectory, facilitating early intervention planning and individualized risk assessment Cardillo et al., [20].

##### Synaptic dysfunction in Alzheimer’s disease

Synaptic dysfunction in Alzheimer’s disease arises from amyloid-β toxicity, tau-mediated microtubule disruption, and neuroinflammatory processes, leading to reduced synaptic plasticity and neuronal loss. These alterations represent some of the earliest detectable changes preceding clinical symptoms. Zhang et al., [110]. Artificial intelligence models trained on neuroimaging, cerebrospinal fluid biomarkers, and proteomic data can identify patterns of synaptic degeneration associated with early cognitive impairment. By integrating molecular and structural indicators, AI systems support preclinical detection and guide targeted preventive interventions aimed at preserving synaptic function Anghelescu et al., [6].

Recent systematic reviews further emphasize the importance of integrating biological biomarkers with machine learning models for early risk prediction and preventive intervention.


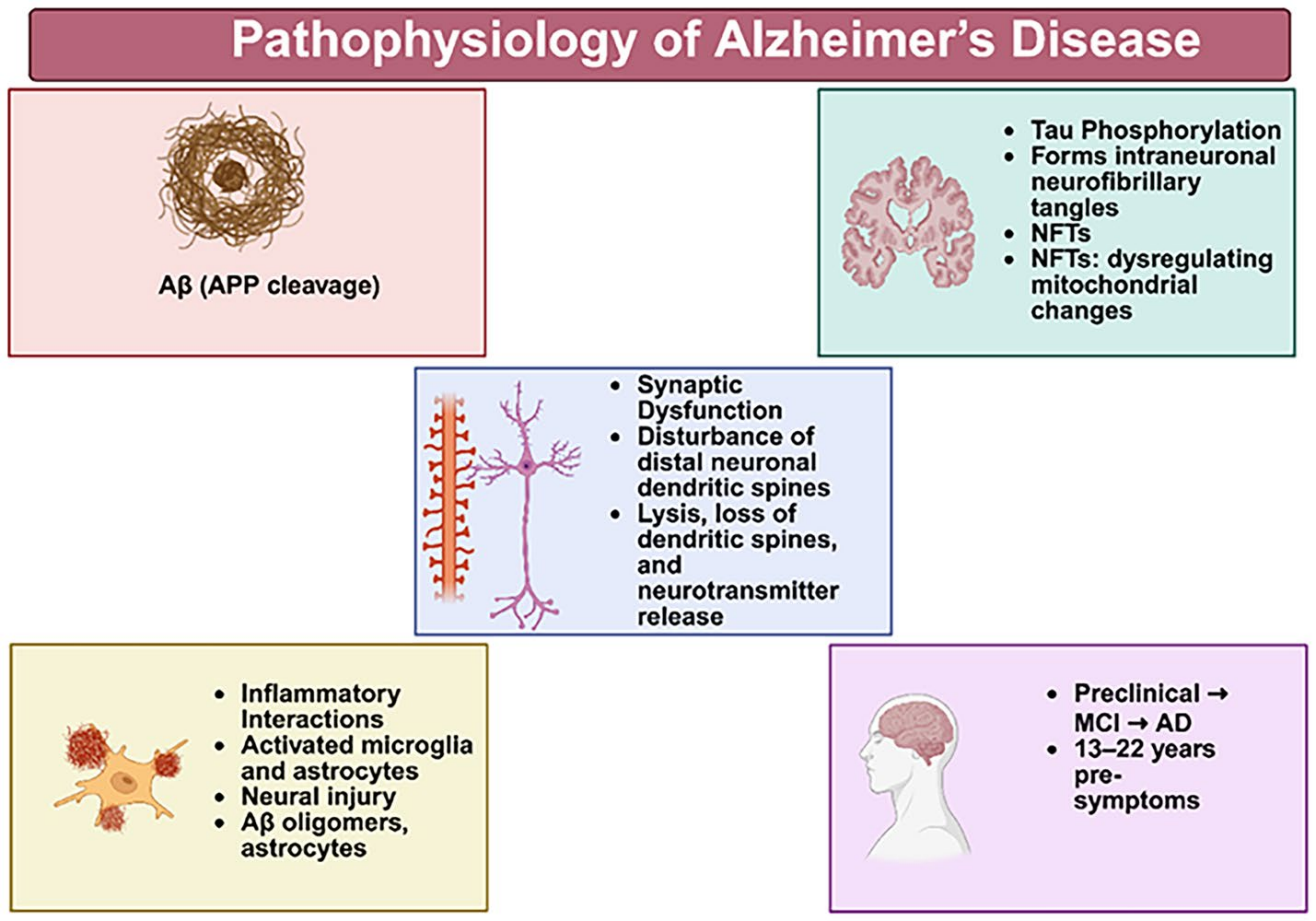


Diagram illustrating the pathophysiology of Alzheimer’s disease, highlighting amyloid-β formation from APP cleavage, tau phosphorylation and neurofibrillary tangle formation, synaptic dysfunction, neuroinflammatory interactions involving microglia and astrocytes and the clinical progression from preclinical stages to mild cognitive impairment and Alzheimer’s disease.

##### Clinical evolution stages

AD advances through stages 0–6: asymptomatic phase with detectable molecular pathology (0–1), mild decline (2), early-stage cognitive impairment (3), early-stage dementia interfering with daily tasks (4), moderate-stage dementia with functional dependence (5), and late-stage dementia with total functional dependency (6). The preclinical and mild cognitive impairment (MCI) phases of AD typically last 5–15 + years longer. MCI affects approximately 8–11% of older people aged 65 years, with a 10–15% annual conversion rate to dementia. Neuropathological changes begin 13–22 years before the onset of symptoms, and post-diagnosis survival lasts between 4 and 8 years. (Alzheimer’s Association, 2025) [113].

### Impact and challenges

Studies on the preclinical stages of dementia have currently made extensive use of the terms mild cognitive impairment (MCI) and its abbreviation, but with varying and contradictory definitions. The clinical term MCI encompasses older adults without dementia who have memory impairment but no obvious disability [77] .Growing knowledge of the cellular and molecular causes of Alzheimer’s disease (AD) has raised hopes for the future creation of treatments that could eventually stop or reverse the course of the illness. As a result, efforts are being made to identify AD as soon as feasible. Advances in structural, molecular, and functional brain imaging have produced techniques that could be helpful in the early diagnosis of AD [45]. Preclinical impairment levels for tasks measuring executive functioning, episodic memory, and perceptual speed are strikingly similar, supporting the idea that several changes in the brain take place before the beginning of clinical disease [10]. The World Health Organization released broadly accepted guidelines on dementia and cognitive decline risk mitigation in 2019. The guidelines give governments, policymakers, healthcare professionals, and other stakeholders a knowledge base to lower the risks of dementia and cognitive decline [92]. Many enzyme inhibitors and antibodies have been studied in people with mild-to-moderate AD over the last 20 years. Regretfully, none of them produced any therapeutic results. It was decided as a result of these failures that a medication cannot reverse or stop the progression of dementia while it is in the mild-to-moderate stage [31].

### Role of early detection and prevention in disease management

The most advanced diagnostic methods for Alzheimer’s disease (AD) currently available are time-consuming (neuropsychological evaluation), costly (neuroimaging), and intrusive (cerebrospinal fluid study). They limit accessibility for frontline screening and diagnostic tools for AD, particularly in preclinical/early phases [55]. The US Food and Drug Administration (FDA) has approved four acetycholinesterase inhibitors to treat Alzheimer’s disease patients with cognitive impairments. These drugs include galantamine hydrobromide (Razadyne; Janssen Pharmaceutica NV, Beerse, Belgium); donepezil hydrochloride (Aricept; Eisai Inc, Woodcliff Lake, New Jersey); rivastigmine tartrate (Exelon; Novartis Pharmaceuticals Corp, Basel, Switzerland); and tacrine hydrochloride (Cognex; Shionogi Inc, Florham Park, New Jersey) [34].


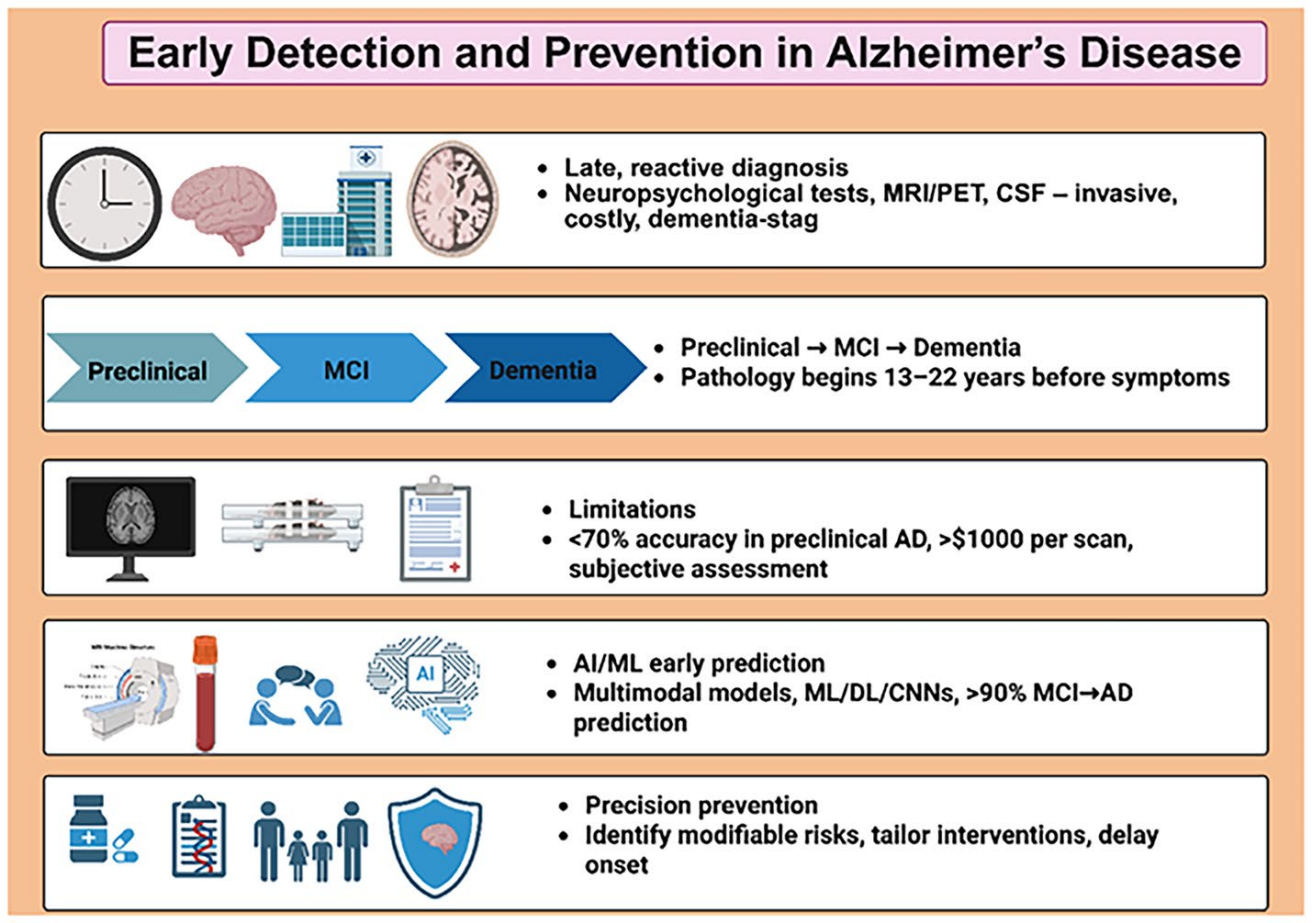


The graphic summarizes current diagnostic limitations, the disease progression continuum from preclinical stages to mild cognitive impairment (MCI) and dementia, and the challenges associated with traditional assessments such as neuropsychological testing and neuroimaging. It highlights the potential of AI/ML-based multimodal prediction models to improve early detection and underscores the role of precision-prevention approaches aimed at identifying modifiable risk factors and delaying disease onset.

#### Bridging the gap: promise of AI/ML

Even with the implementation of these tools, conventional diagnostic methods are often inadequate for early identification due to low sensitivity for subtle alterations (e.g., accuracy below 70% during preclinical stages), high associated cost (with neuroimaging exceeding $1,000/session), clinical invasiveness, and reliance on subjective human assessment, resulting in delaying intervention at the stage when neuroprotection is most effective [71, 76]. Artificial intelligence (AI) and machine learning (ML) mitigates these limitations by facilitating noninvasive, scalable analysis of multimodal data (e.g., MRI, genetics, wearables), achieving more than 90% accuracy in predicting MCI-to-AD conversion several years in advance, thereby enabling precision prevention and bridging the translational gap from pathological insights to actionable clinical care [71, 76].

### AI & ML as transformative tools in healthcare

New approaches in artificial intelligence (AI) have the potential to completely transform drug development, from preliminary research to pre-clinical and clinical phases. The AI models comprise algorithms based on network, deep learning, and machine learning (ML). Large-scale biomedical data has shown that AI-based models are quicker and more efficient than conventional biological experiments [82]. Recent advances in artificial intelligence have made it feasible to use machine learning for diagnostic purposes. This has sparked a lot of research interest in a wide range of disorders, including the detection of diabetes, Parkinson’s disease, peripheral artery disease, heart disease, and many more. Machine learning-based techniques for Alzheimer’s disease diagnosis have also gained attention recently. Several important test results and volume measurements, such as the Mini-Mental State Examination, Clinical Dementia Rating, AD Cognitive Scale Score, Rey’s Auditory Verbal Learning Test, and the volumes of the brain, hippocampal, ventricles, entorhinal cortex, middle temporal lobe, intracranial, and fusiform gyrus, are used to diagnose Alzheimer’s disease [21]. The majority of recent research, however, has made use of brain imaging technology that is impractical for clinical settings. Machine learning models have been used to predict AD in a pilot study that included 133 patients with AD, 21 patients with MCI, and 31 healthy controls. One of the agonists at the NMDARs is D-glutamate. The best models for identifying MCI and AD seemed to be the random forest model and the naïve Bayes model [22]. Technology can help with dementia care, which will lessen the strain on caregivers. Research has investigated the use of robotics, virtual reality, music technology, the Internet of Things, and virtual assistants to help people with dementia. To be more precise, intelligent voice assistants have been proposed as a possible help to caretakers, who are typically elderly persons with little experience with technology [46].

## Current landscape of Alzheimer’s detection and prevention

Clinical evaluation, cognitive testing, and neuroimaging are the primary focuses of traditional Alzheimer’s disease (AD) diagnostic techniques. But in terms of precise diagnosis and early detection, these approaches are severely limited. Below is a summary:

### Clinical evaluation and cognitive testing

The most often used test for dementia screening is the Mini-Mental State Examination (MMSE). Since the MMSE’s intellectual property rights were transferred to Psychological Assessment Resources in 2001, its usefulness and accessibility have decreased. Nonetheless, over 40 additional tests, many of which are publicly accessible, are available for dementia screening in healthcare settings. These include the MiniCog test, the General Practitioner Assessment of Cognition (GPCOG), the Informant Questionnaire on Cognitive Decline in the Elderly (IQCODE), the Addenbrooke’s Cognitive Examination–Revised (ACE-R), and others. Since the MMSE is a private tool and costs money, its diagnostic performance has not been thoroughly assessed and combined for comparative analysis [95].

### Neuroimaging techniques

Computed tomography (CT), magnetic resonance imaging (MRI), and positron emission tomography (PET) are three strong imaging modalities that have made it possible to examine anatomical and functional abnormalities in a variety of central nervous system (CNS) illnesses [32]. Previously, imaging technology including computed tomography (CT) and MRI, was solely used to rule out reasons of cognitive deterioration that might be treated surgically. By spotting distinctive patterns (signatures) of structural and functional brain changes, it can now contribute to the positive confirmation of a clinical diagnosis of AD [47]. MRI may be a better way to differentiate vascular or mixed dementia from Alzheimer’s disease and other disorders than CT, according to comparative analyses. However, the confidence intervals on calculated diagnostic odds ratios were quite large [15].

After 2004, PET imaging showed an annual increase rate of 57%. There are numerous uses for PET scanning in the treatment of neuropsychiatric disorders, mobility problems, and neurodegenerative diseases. Before alterations are seen on structural imaging, anomalies on PET imaging can be identified by looking at metabolism, enzyme activity, protein accumulation, and receptor binding [90].

### CSF biomarker analysis

The translucent body fluid that fills the ventricular system and subarachnoid space around the brain is called cerebrospinal fluid, or CSF. When it comes to finding biomarkers for the prognosis of neurodegenerative diseases, CSF is arguably the most informative fluid. In particular, CSF biomarkers would be useful. The oldest and most thoroughly researched biomarkers in CSF are tau and Aβ. Amyloid plaques, neurofibrillary tangles, and other characteristic lesions of AD are associated with both proteins [67]. The Alzheimer’s Association and the National Institute on Aging commissioned a work group to produce the biomarker-based biological categorization known as the Amyloid/Tau/Neurodegeneration (A/T/N) system in 2018. The study of cerebrospinal fluid (CSF) biomarkers for Alzheimer’s disease has grown significantly over the past 20 years [68].

### Genetic testing

For late-onset Alzheimer’s disease, variants of the apolipoprotein E (APOE) gene have the highest hereditary risk. An amino acid sequence difference between positions 112 and 158 distinguishes the three isoforms of a secreted 299 amino acid protein (apoE2, apoE3, and apoE4) that are encoded by APOE [66]. ApoE4 appears to work through both Aβ-dependent and Aβ-independent pathways to raise the risk of AD and cognitive impairment. Aβ synthesis, aggregation, and clearance are variably regulated by ApoE isoforms. Lastly, ApoE isoforms play distinct functions in preserving vascular health, which are important considering that vascular health abnormalities are closely linked to AD. Although it is a difficult task, determining this gene contributes to the pathophysiology of AD may help fight the disease Liu et al. [62].

### Blood-based biomarkers

There are currently no FDA-approved blood tests for AD, most are clinically available as laboratory-developed diagnostics. Crucially, there is a great deal of heterogeneity in these tests’ validity and performance. Precivity AD, the first clinically available test for AD, was provided by C2N Diagnostics in St. Louis, Missouri, USA. It calculated a risk score for amyloid PET positive based on age, plasma Aβ42/Aβ40, and an apolipoprotein E prototype [8]. An economical and efficient method of improving the usefulness of CSF and imaging biomarkers is to use blood-based AD biomarkers. The ability of these biomarkers to distinguish between individuals with moderate cognitive impairment (MCI) who developed AD and those who did not further suggests that they may also have predictive significance [40].

### Comparative analysis: traditional vs. AI/ML approaches

**Table 1:** Comparative table (Traditional vs. AI/ML)

S.No.	Criteria	Traditional approach	AI/ML-based approach
1.	METHODS	Clinical and cognitive techniques including neuroimaging techniques (PET, MRI), biomarker analysis, genetic testing, and recently discovered blood test for AD.	Traditional ML (supervised ML model, unsupervised ML model, semi-supervise ML model), and Deep learning ML (artificial neural networks, convolutional neural networks, and deep-belief neural networks)
2.	OBJECTIVITY	Clinical and cognitive assessments are subjective in nature and rely on clinician expertise, patient/informant report, and interpretation of reports which is highly variable.	The diagnosis is based on mathematical algorithms and learned patterns, making it highly objective, reproducible, and less variable.
3.	DIAGNOSTIC ACCURACY	These methods are inconsistent especially limited in early-stage AD and differentiating from other dementias. Biomarker changes may precede symptoms but are not widely accessible.	Superior accuracy and predictive power demonstrated in many studies for classification. There is high-potential for early/pre-symptomatic detection by identifying subtle, complex patterns in data.
4.	SCALABILITY AND COST	Often high-cost (PET scans, specialised CSF collection) and low-throughput, with limited accessibility.	Potential for high-throughput is evident with lower cost and higher scalability, especially with blood based, neuroimaging, genetic, biochemical, and speech analysis, making it more practical for screening.
5.	DATA SOURCE	Primarily a single modality is present; cognitive test score, a single neuroimaging scan, or individual biomarker levels.	Multimodal data integration is present where vast amounts of data from neuroimaging, biomarkers, cognitive and speech analysis, and genetics are combined and analysed collectively, while compensating for the loopholes left during scans via techniques like Generative AI.
6.	KEY CHALLENGE	Late diagnosis, dependence on expert interpretation, invasiveness (CSF, blood), high cost with limited access, and inability to synthesise multimodal, complex data.	Data requirements, high sensitivity and dependence on nature of data, model interpretability (black box), lack of clinical validation, and regulatory approval for widespread use.

The comparison presented is based on representative studies and general trends reported in the literature. Reported performance of AI/ML-based approaches varies substantially depending on dataset size, population characteristics, and validation strategy. Most studies rely on retrospective or publicly available datasets and employ internal validation, with limited external or prospective evaluation (Table 1).


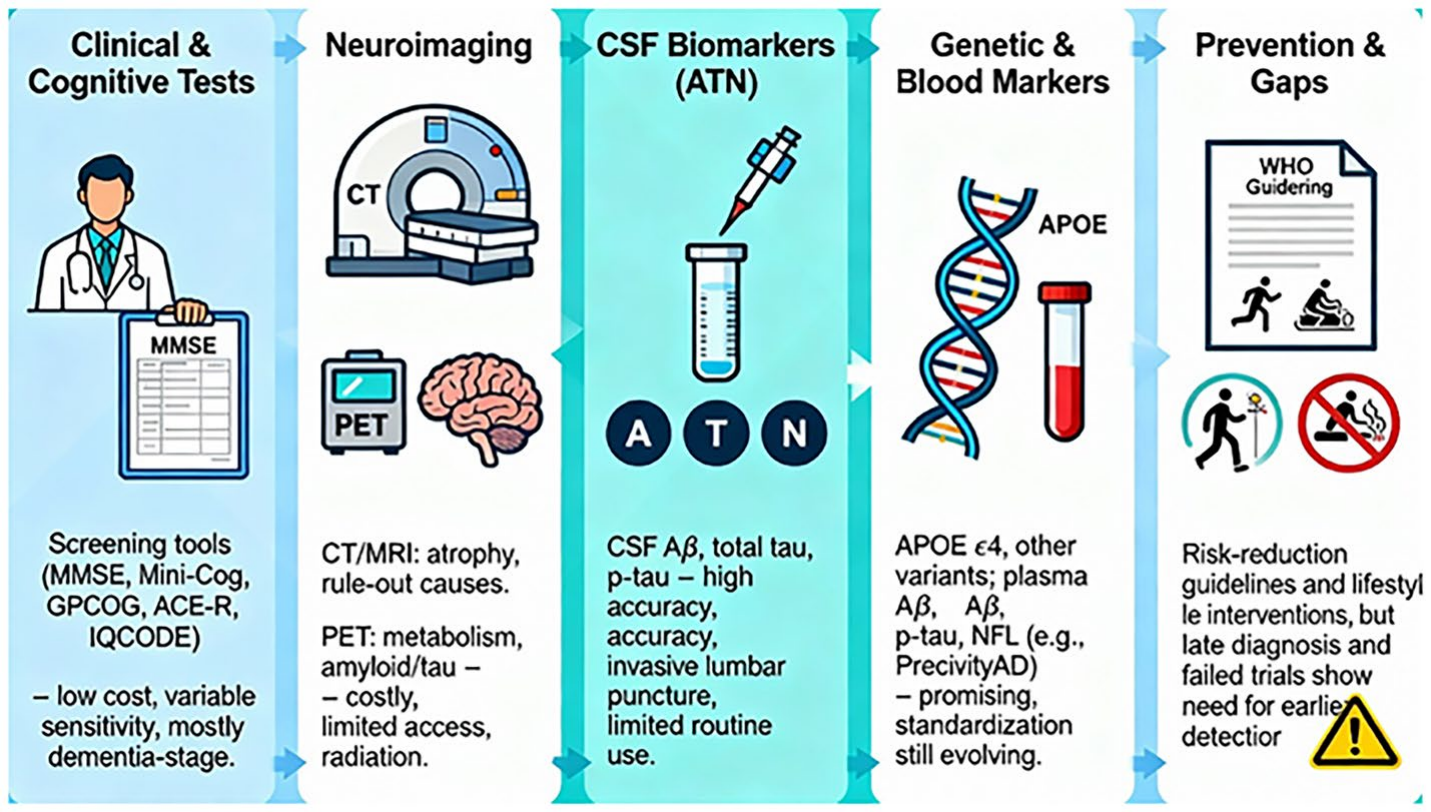


## Role of artificial intelligence and machine learning

### Fundamentals of AI and ML

Artificial intelligence and machine learning techniques are increasingly applied in Alzheimer’s disease research to analyze complex neuroimaging, biomarker, genetic, and clinical datasets. These methods support early detection, risk stratification, and prediction of disease progression by identifying patterns that are not readily detectable through conventional statistical approaches Kaul et al. [50].

### Methodological limitations and reproducibility challenges

Despite rapid advances in artificial intelligence applications for Alzheimer’s disease, many published models exhibit methodological weaknesses that limit clinical translation. Overfitting remains a major concern, particularly in studies using small or homogeneous datasets, where models perform well during internal validation but fail to generalize to independent populations.

Data leakage, arising from inappropriate preprocessing or feature selection prior to data splitting, has also been reported in several studies, leading to inflated performance estimates. Furthermore, inconsistent reporting of training procedures, hyperparameter tuning, and missing data handling reduces reproducibility across research groups.

External validation using geographically and demographically diverse cohorts is rarely performed, and prospective clinical evaluation is uncommon. In addition, dataset shift caused by evolving clinical practices, imaging protocols, and population characteristics further undermines long-term model reliability. These limitations highlight the need for transparent reporting standards, standardized benchmarking, and rigorous multi-center validation before widespread clinical deployment Weiner et al. [103].

### Machine learning techniques in AD

ML methods that have been applied in dementia research can be divided broadly into two categories: (1) traditional ML and (2) deep learning (DL) [19].

#### Traditional ML

##### Supervised learning

In order to learn a function (f), a supervised machine learning algorithm uses the training data to map specific input variables or features (X) into an output or target (Y). Supervised machine learning platforms often use “labeled” training data sets to provide either a qualitative or quantitative result. One important aspect of this approach is that the training phase’s data evaluation is labeled, which enables the ML model to eventually mimic the expert’s input data. Consequently, the machine learning model can differentiate an unknown input by using its previous training parameters [83].

In the medical field, this could mean training a model to associate a person’s traits (height, weight, and smoking status) with a particular result (for instance, the onset of diabetes within five years) [89]. This is particularly useful when diagnosis of a neurodegenerative disease is expected to be done based on the persons present traits/ condition, including speech and cognitive impairments, lifestyle habits, genetic factor amongst others. The most widely utilized machine learning techniques for patient classification and identifying likely progression from MCI to AD dementia were support vector machine (SVM) based techniques. SVM is a supervised machine learning algorithm that was utilized in 60 of the 116 research. It has shown its value in neuroimaging-based applications, particularly in the categorization of upcoming clinical results [36].

##### Unsupervised learning

Here, “supervised” means that the algorithm’s training process is overseen by the presence of the right responses, meaning that we are aware of the desired result. Nonetheless, the learning process is referred to as unsupervised when there is only a set of variables and no matching output variables (i.e., the data are unlabeled). Therefore, in unsupervised learning, the learning algorithm is left to find the structures in the datasets since there are no right answers for the training process to use [4].

A viable way to find, measure, and describe clinical AD subpopulations is through unsupervised machine learning approaches. Clustering and association rule mining are examples of unsupervised machine learning methods that conclude from data without using predetermined or labeled results. The current study used clustering algorithms to find new subpopulations of Alzheimer’s patients using data from the ADNI database, such as biomarker values, clinical cognitive assessment scores, basic MRI imaging features, and patient socio-demographics. Finding baseline characteristics of subpopulations at the time of initial diagnosis was specifically the emphasis, as this could aid in pre-classifying individuals for improved stratification and representation in AD therapeutic trials [81]. Specification of such characteristics would prove useful in the future for AD diagnosis at the pre-symptomatic stage and reduce risk of deterioration of condition.

##### Semi-supervised learning

Halfway between supervised and unsupervised learning is semi-supervised learning (SSL). Unlabelled data, or data from MCI people for whom a trustworthy future diagnosis cannot be established, is used by SSL techniques. In our instance, there are comparatively few unlabelled and labelled data accessible, although in normal SSL applications in machine learning (speech recognition, text classification, etc.), there should be a large amount of available unlabelled data. Thus, it is critical to investigate the potential bottlenecks of SSL techniques as well as when semi-supervised learning is beneficial, i.e., when unlabelled data can increase classification accuracy Modern Care for Patients with Alzheimer Disease: Rationale for Early Intervention Moradi et al. [70].

In a semi-supervised approach, in addition to the labels and feature vectors from the labelled data, the learning process also uses feature vectors from unlabelled data. Finding an estimate of the data dispersion in the feature space will be made easier with the help of the information gleaned from this unlabelled data. One crucial presumption must be made before executing a semi-supervised learning algorithm: if two dataset members are situated in a dense area and are near to one another in the feature space, their labels will likewise be near to one another [53].

#### Deep learning

Deep learning architectures, including convolutional neural networks, recurrent networks, and transformer-based models, are widely used in Alzheimer’s disease research for automated feature extraction from neuroimaging, speech, and multimodal data. These models enable high-dimensional pattern recognition and improve the detection of subtle pathological changes associated with early neurodegeneration Bucholc et al. [19].

##### Artificial neural networks

A collection of algorithms called a neural network is utilized to identify patterns and information in the MRI dataset. Therefore, we extend the conventional neural network with additional layers for deep learning. The number of layers varies from 10 to 100, depending on the computation network. Information is stored by each layer’s neurons and then transmitted to the forward neurons. The MRI pictures are used to retrieve the concealed information when this data moves from the network. Typically, lower layer neurons gather the unprocessed data [7].

Recent studies have shown that the final established ANN, which contains a variety of information such as biomarkers, neuropsychological functions, and epidemiological factors, has a high level of diagnostic efficiency and precision. It can be considered a practical, low-cost method for AD screening and diagnosis [101].

##### Convolutional neural networks

Classic neural networks are comparable to convolutional neural networks, which draw inspiration from the human visual system. This architecture was specifically created with the explicit premise that the raw data are two-dimensional (pictures), which allows us to encode certain attributes and minimize the number of hyperparameters. The CNN topology enhances generic feed-forward back propagation training by reducing the number of parameters that need to be learned through the use of spatial correlations [87]. A CNN-based deep learning model is suggested for MRI scan-based Alzheimer disease identification.

The automatic identification of AD phases by MRI scans has been surpassed by a number of pre-trained CNN models. Successful MRI analysis applications include the pre-trained AlexNet, deepNN, ResNet-50, VGG11, ResNet-34, SqueezeNet, DenseNet, and InceptionV3. Compared to a system that uses a single structure pre-trained on MRI images, several pre-trained networks on a large scale with MRI may provide potentially important structural information for differentiating the AD phases [88].

The model proposes an automated framework that incorporates Convolutional Neural Networks (CNNs), which markedly enhanced the accuracy, sensitivity (true positive rate), and specificity of Magnetic Resonance Imaging-driven Alzheimer’s Disease diagnosis. The utilization of AI techniques for the detection of Alzheimer’s Disease has yielded favourable outcomes with heterogeneous datasets, including Magnetic Resonance Imaging (MRI), Electroencephalography (EEG), Positron Emission Tomography (PET), Magnetoencephalography (MEG) and sensor-generated data. (Lin et al., 2018a) [115].

##### Deep belief networks

A generative model called a Deep Belief Network (DBN) makes use of several processing layers to identify intricate patterns and abstractions in data. Individually trained Restricted Boltzmann Machines (RBMs) are piled on top of one another to form this system. Unsupervised training is used to train the RBMs in a DBN, with an unsupervised stage at the start of the training process [87].

### Advantages of AI/ML in AD prevention

For Alzheimer’s disease (AD) and its prodromal stage, mild cognitive impairment (MCI), computer-aided diagnosis (CAD) has gained popularity in recent years. CAD technologies now in use, however, frequently overfit data and have low generalizability. According to rate distortion (RD) theory and an extreme learning machine (ELM) model, we developed a sparse-response deep belief network (SR-DBN) model in this study to differentiate between AD, MCI, and normal controls (NC), applications of AI tools discussed in table-2 [87].

**Table:** 

Application	Description	AI tools used	References
Early Diagnosis	Multi-modality imaging, including MRI, FDG-PET, amyloid-PET, and the recently introduced tau-PET, which provide distinct but complementary information, makes it possible to diagnose AD and provide a prognosis (likelihood of converting to AD) at these early stages, which are difficult tasks.	Machine Learning, Deep Learning and Image Processing	Liu et al., [63]
Cognitive Assessment	Early-stage dementia is known to impair speech and language ability, with symptoms including aphasia, pauses, decreased vocabulary, and other language difficulties.	Machine Learning Deep Learning Natural Language Processing	Li et al., [58]
Identification of Biomarker	Numerous biological dimensions of biomarkers, such as neuroimaging, electrophysiological, genetic, protein, metabolic, sleep and digital indicators, have been studied in dementia patients. The diagnosis of dementia may be improved by biomarkers from cerebrospinal fluid (CSF) and minimally invasive biological fluid collection, such as blood, saliva, tears, and urine.	Supervised Learning and Unsupervised Learning	Winchester et al., [105]
Drug repurposing	A novel AI-driven drug-repurposing method, Deep Drug, to identify a lead combination of approved drugs to treat AD patients.	Graph Neural Networks, Signed Directed Graph Neural Networks, Computational Drug Design and Machine Learning	(V. O. K. Li et al., [60]
Drug Discovery and Clinical Trials	More general understanding of their application and strong consensus guidelines on their execution can improve drug development and clinical trials in AD and lower the risk associated with novel clinical development approaches.	Predictive analysis and Machine Learning	Doherty et al., [29]

## Data sources and technologies

### Clinical imaging data (MRI, PET SCANS)

In the field of Alzheimer’s disease research and diagnosis, Multimodal Imaging Analysis is a state-of-the-art method that is revolutionizing our understanding of this intricate neurodegenerative illness. In order to provide a comprehensive and detailed representation of the illness, this novel approach combines information from a variety of imaging modalities, including Computed Tomography (CT), Positron Emission Tomography (PET), and Magnetic Resonance Imaging (MRI) [108].

The computerized x-ray imaging process known as “computed tomography,” or “CT,” involves aiming a narrow x-ray beam at a patient and rapidly rotating it around the body to produce signals that the machine’s computer processes to create cross-sectional images, or “slices,” of the body. Compared to traditional x-rays, these slices known as tomographic images contain more comprehensive information. Following the collection of multiple consecutive slices by the machine’s computer, these slices can be digitally “stacked” to create a three-dimensional image of the patient, making it simpler to locate and identify fundamental components as well as potential tumors or anomalies [43].

MRI is a non-invasive imaging technique that creates detailed, three-dimensional anatomical images without using harmful radiation. Disease detection, diagnosis, and therapy monitoring are among its frequent uses. Its foundation lies on advanced technology that stimulates and senses changes in the direction of the protons’ rotating axis in the water that constitutes biological tissues. They are different from computed tomography (CT) in that they do not employ x-rays, which are harmful ionizing radiation. Compared to standard x-rays and CT scans, MRI provides a far clearer image of the brain, spinal cord, and nerves as well as muscles, ligaments, and tendons. MRI can identify tumors and aneurysms in the brain as well as distinguish between white and grey matter. However, MRI is more costly than CT or x-ray imaging [44].

Positron emission tomography (PET) is a method used to quantify physiological function by examining radiolabelled medicines, blood flow, metabolism, and neurotransmitters. The quantitative analyses provided by PET enable the monitoring of relative changes over time as a disease process progresses or in response to a particular stimulus. After a little quantity of a radioactive tracer is injected into a peripheral vein, the procedure relies on the measurement of radioactivity released. The tracer is often tagged with oxygen-15, fluorine-18, carbon-11, or nitrogen-13 and is given intravenously. In comparison to computed tomography, the overall radioactive dose is comparable [13].

The earliest imaging methods utilized in AD were computed tomography (CT) and magnetic resonance imaging (MRI), although these methods were not employed to diagnose AD early on, but rather to rule out other causes of dementia. In the future, imaging methods were used to support the clinical diagnosis of AD. These methods concentrated on the degeneration and neuronal damage associated with AD. These days, imaging techniques are mostly used to detect neurodegeneration or amyloid buildup [97]. The ability to visualize and measure the aberrant amyloid beta (Aβ) load in the living brain makes Positron Emission Tomography (PET) imaging essential for monitoring the course of the disease and assessing the effectiveness of anti-amyloid treatments. Realistic synthetic images can be produced by generative artificial intelligence (AI), which can also learn intricate data distributions [17].

### Electronic health records and patient aggregation

Recent advancements have made the use of machine learning for diagnostics a viable option with considerable research interest in a wide range of illnesses, including the detection of diabetes, Parkinson’s disease, peripheral artery disease, heart disease, and many more. The ability of machine learning-based techniques to learn from massive datasets makes them extremely promising for providing solutions with major societal impacts. Machine learning-based techniques for Alzheimer’s disease diagnosis have also gained attention recently. A number of important test results and volume measurements, such as the Mini-Mental State Examination, Clinical Dementia Rating, AD Cognitive Scale Score, Rey’s Auditory Verbal Learning Test, and the volumes of the brain, hippocampal, ventricles, entorhinal cortex, middle temporal lobe, intracranial, and fusiform gyrus, are used to diagnose Alzheimer’s disease, datasets summary discussed in table-3.

**Table:** 

Dataset	Modality	Participents	Application	References
ADNI (Alzheimer’s Disease Neuroimaging Initiative)	Neuroimaging methods (MRI, PET) and biomarkers	Normal individuals, persons with MCI, elderly patients and subjects with mild AD.	Formed by a group of medical facilities and universities in the US and Canada, it aims to create improved techniques that will result in consistent standards for gathering longitudinal, multisite MRI and PET data; create an easily accessible data repository that details changes in brain structure and metabolism over time while simultaneously gathering clinical, cognitive, and biochemical data; create techniques that will optimize the ability to assess treatment effects in clinical trials; and test a number of hypotheses based on clinical and biomarker data.	Petersen et al., [79]
OASIS (Open Access Series of Imaging Studies)	Neuroimaging data (cross-sectional MRI)	Subjects with and without dementia and AD.	The goal of the initiative is to make brain neuroimaging data sets publicly accessible to the scientific community. by assembling and making neuroimaging data sets publicly available.	Oostveen and Lange, [98]
AIBL (Australian Imaging, Biomarkers & Lifestyle Study)	Neuroimaging data (MRI, amyloid PET, tau PET), fluid & genetic biomarkers, cognitive tests, lifestyle and dietary factors.	Healthy individuals, subjects with mild cognitive impairment, and patients diagnosed with AD	AIBL seeks to better understand the etiology, early clinical presentation, and diagnosis of AD as well as the lifestyle and dietary factors that contribute to its progression, using baseline cohort cross-sectional data. The connections between cognition, brain Ab load, structural alterations in the brain, biomarkers, and lifestyle will be further uncovered by these cross-sectional studies.	(Ellis et al., 2010) [114]
NAAC (National Alzheimer’s Coordinating Center)	Data items specific to its patients enrolled in Alzheimer disease centres. (clinical and medical history, physical and chemical examination, cognitive ability analysis, behavioural assessment, diagnosis, treatment)	The National Institute on Aging funds 29 ADCs, and NACC is in charge of creating and managing a database of patient-specific data gathered from these centres. Every center relevantly gathers and sends a minimum dataset to NACC.	Longitudinal Analysis Disease stage prediction	(Beekly et al. [12])

## Early detection methods

### AI-based risk assessment models

#### Conditional restricted boltzmann machine

A research group effectively simulated patients’ illness progression and formulated a tailored model for prediction of disease progression employing a Conditional Restricted Boltzmann Machine model (CRBM) and a dataset comprising 18 months of longitudinal clinical records (44 variables were gathered) from 1,909 patients with Alzheimer’s Disease (AD) or Mild Cognitive Impairment (MCI). This approach forms part of a wider AI-enabled risk prediction system that offer an innovative approach for clinical management and prevention of Alzheimer’s disease (AD), as AI-based detection of pathophysiological alterations arise well before clinical manifestations emerge, advocating the significance for early interventions (Fisher et al., 2019; [91]).

#### Multi-omics & GWAS frameworks

Through the use of a web-based AI framework which incorporates multi-omics datasets and Genome-Wide Association Studies (GWAS) results, the researchers effectively discovered 103 genes implicated with Alzheimer’s Disease risk. Moreover, the study revealed three medications, including pioglitazone, were strongly correlated with a considerable decrease in occurrence of Alzheimer’s Disease. This framework underpins the elucidation of genetic and environmental contributors to disease risk, in accordance with Artificial Intelligence’s role in projecting disease trajectories with high precision and facilitating prevention strategies (Stern & Linial, 2025).

#### UKB-dementia risk prediction model

A novel framework or innovative model was established for Alzheimer’s Disease risk prediction, UK Biobank-Dementia Risk Prediction Model, which employed Machine Learning (ML) approaches to a dataset of 366 features (hereditary and environmental determinants), pinpointing 10 critical predictive factors such as age and Apolipoprotein E ε4 (ApoE ε4). The model accurately forecasted Alzheimer’s Disease risk over periods of 5 years, 10 years, and beyond. This apporach illustrates AI-driven risk forecasting, providing a framework for reducing risk, postponing disease onset, or attenuating pathological progression of Alzheimer’s Disease [91].

#### Deep learning multimodal models

A Deep Learning (DL) model was constructed which incorporates multi-modal datasets and interaction effects. This approach reliably forecasted the progression from Mild Cognitive Impairment (MCI) to Alzheimer’s Disease (AD) within four years, with predictive accuracy of 92.92%. Estimating disease conversion from Mild Cognitive Impairment to Alzheimer’s Disease has gained prominence as a vital research area, and this model extends existing frameworks that combine longitudinal and multidomain datasets.

#### Random forest models

This model leveraged data of Magnetic Resonance Imaging (MRI) in conjunction with supervised learning and applied Random Forest algorithms to identify a novel biological marker for Alzheimer’s Disease (AD), substantially enhancing diagnostic reliability and creating the foundation for early therapeutic measures. A subset of Artificial Intelligence methods, including Machine Learning (ML) and Deep Learning (DL) models, have demonstrated potential in interpreting and combining multi-source data for establishing predictive models aimed at Alzheimer’s Disease diagnosis. (Rathore et al., 2017)

#### Fusion-loss deep learning models

This approach introduced a Deep Learning (DL) model optimized using a fusion loss function to accurately and efficiently classify the stages of Alzheimer’s Disease based on MRI images. The model delivers high classification precision and robust capability in monitoring disease trajectories, facilitating clinical decision-making for physicians. The implementation of AI-based approaches for identifying Alzheimer’s Disease has yielded positive results with heterogeneous data sources, such as Magnetic Resonance Imaging, Electroencephalography, Positron Emission Tomography, and sensor data, overview of case studies discussed in table-4. 

### Case studies: early prediction of AD progression

**Table:** 

S.No.	Case theme	Study type	Model type	Accuracy	Years before onset predicted	Refs.
1.	Ensemble deep transfer models for predicting long-term cognitive decline to Alzheimer’s disease.	Alzheimer’s disease neuroimaging initiative (ADNI) datasets.	Ensemble deep transfer models	0.85 & F-1 score of 0.86.	Forecasted conversion from normal cognitive decline to Alzheimer’s disease up to 10 years in advance	Aghaei and Moghaddam, [3]
2.	Deep learning-based image processing for Early Alzheimer’s Disease.	Open Access Series of Imaging Studies (OASIS) MRI data.	Deep learning-based model	88% accuracy	Forecasted early-stage Alzheimer’s Disease prediction up to 5 years pre-symptoms.	Kavitha et al., [51]
3.	Deep Neural Network-Based Computational Modeling of Dementia	OASIS (Open Access Series of Imaging Studies) datasets.	Deep Neural Network	92% accuracy	Forecasted Alzheimer’s Disease trajectories (up to 5 years pre-symptoms).	Kavitha et al., [51]
4.	Bidirectional Gated Recurrent Unit Deep Learning for predicting Mild Cognitive Impairment (MCI) to conversion in Alzheimer’s Disease.	Medicare claims data	Bidirectional Gated Recurrent Unit Deep Learning	Area Under the Receiver Operating Characteristic Curve (AUC-ROC) of 0.833, Area Under the Precision-Recall Curve (AUC-PR) of 0.856, and F1-score of 0.71.	Forecasted MCI to Alzheimer’s Disease progression over 5 years	Abdelhameed et al., [1]
5.	Support Vector Machine (SVM) framework from Whole-Brain Magnetic Resonance Imaging (MRI) Volumes for early detection of Alzheimer’s Disease.	ADNI (Alzheimer’s Disease Neuroimaging Initiative) datasets.	Support Vector Machine (SVM) framework for MRI	75% accuracy	Prediction of MCI to Alzheimer’s Disease progression up to 3 years before onset.	Grueso and Viejo-Sobera, [37]
6.	Support Vector Machine (SVM) for early detection of Alzheimer’s Disease using Multimodal Magnetic Resonance Imaging (MRI) and Positron Emission Tomography (PET) .	ADNI (Alzheimer’s Disease Neuroimaging Initiative) datasets.	Support Vector Machine (SVM) with MRI and PET	79% accuracy	Forecasted progression of MCI to AD up to 3 years before onset.	Grueso and Viejo-Sobera, [37]
7.	Convolutional Neural Network (CNN) approach for early Alzheimer’s detection using Multimodal data.	Multimodal datasetes	Convolutional Neural Network (CNN)	Mean accuracy of 78.5% (up to 97.3% in sub-studies)	Forecasted MCI to AD conversion up to 3 years before onset.	Bae et al., [11]
8.	Structural MRI Grading Biomarker for MCI to Alzheimer’s Disease prediction.	ADNI (Alzheimer’s Disease Neuroimaging Initiative) datasets.	Structural MRI Grading Biomarker	81.9% accuracy	Forecasted conversion of MCI to AD up to 3 years before onset.	Tong et al., [93]
9.	Deep Learning analysis of Brain Metabolism and Amyloid Imaging.	ADNI (Alzheimer’s Disease Neuroimaging Initiative) datasets.	Deep Leaning Networks	AUC of 0.89	Cognitive decline prediction up to 2 years pre-symptoms.	Choi and Jin, [24]
10.	Integrated Functional Magnetic Resonance Imaging (FMRI) and Structural Magnetic Resonance Imaging (SMRI) for predicting conversion of MCI to AD.	Resting state FMRI and SMRI	Integrated Functional Magnetic Resonance Imaging (FMRI) and Structural Magnetic Resonance Imaging (SMRI)	85.7% accuracy	Forecasted Alzheimer’s Disease progression up to 3 years before onset.	Hojjati et al., [41]
11.	Deep Learning analysis of Hippocampal MRI for Alzheimer’s Disease Progression.	ADNI (Alzheimer’s Disease Neuroimaging Initiative) datasets.	Deep Learning Networks	AUC of 0.92 for Alzheimer’s Disease progression	Forecasted up to 2 years pre-symptoms.	Li et al., [56]
12.	Convolutional Neural Network (CNN) framework for early Alzheimer’s prediction using MRI.	ADNI (Alzheimer’s Disease Neuroimaging Initiative) datasets.	Convolutional Neural Network (CNN)	88.2% accuracy	Forecasted MCI to AD conversion up to 3 years before onset.	(Lin et al., 2018b) [115]
13.	Hybrid Convolutional Neural Network-Support Vector Machine (CNN-SVM) model on Alzheimer’s Disease Neuroimaging Initiative data for MCI to AD prediction.	MRI-based feature extraction (hippocampi, temporal lobes) and multimodal datasets.	Hybrid Convolutional Neural Network-Support Vector Machine (CNN-SVM) model	88% accuracy and AUC of 0.95 for MCI progression to AD	Forecasted MCI progression to AD (up to 2 years follow-up).	(Wang et al., 2022a) [116]
14.	Japanese ADNI-based Hybrid Convolutional Neural Network-Support Vector Machine (CNN-SVM) Cross-Cohort approach for early detection of Alzheimer’s Disease.	North ADNI, Japanese Alzheimer’s Disease Neuroimaging Initiative (J-ADNI)	Hybrid Convolutional Neural Network-Support Vector Machine (CNN-SVM) Cross-Cohort approach	84% accuracy and AUC of 0.91	Forecasted Mild Cognitive Impairment conversions to Alzheimer’s Disease up to 3 years follow-up.	(Wang et al., 2022b) [116]
15.	Temporal Graph Network for modelling Dynamic Alzheimer’s Disease Progression.	ADNI (Alzheimer’s Disease Neuroimaging Initiative) datasets.	Temporal Graph Network	AUC of 0.8090 (0.8807 with neuroimaging)	Forecasted onset of Alzheimer’s Disease progression dynamics	(Aqil et al., 2024) [117]

Most studies summarized in this table were conducted on single-center or publicly available datasets, primarily ADNI and OASIS. Sample sizes and class distributions varied considerably, and external validation was uncommon. Reported performance metrics should therefore be interpreted with caution.

## Preventive interventions guided by AI

### Identification of modifiable risk factors

Artificial Intelligence (AI) serves a central role in pinpointing risk factors amenable to intervention for Alzheimer’s Disease (AD) and dementia, facilitating targeted prevention approaches. A diverse set of changeable risk factors for dementia have been ascertained, with substantial controversy surrounding their interplay with genetic risk factors, which AI can aid in elucidating using advanced data analysis methods [72].

By integrating extensive multimodal datasets and high-throughput technologies, unrestricted by the scales of datasets. Through hierarchical predictive models and genomic subtyping, AI discovers biological markers, incorporating chargeable factors such as molecular and lifestyle determinants linked to the risk of dementia. These frameworks combine diverse datasets, including Cerebrospinal Fluid (CSF) biomarkers (e.g., Amyloid Beta [Aβ], Total Tau [t-tau], Phosphorylated Tau [p-tau]) and Positron Emission Tomography (PET) imaging, to elucidate risk factors such as cardiovascular and environmental factors, amenable to intervention for dementia prevention [26, 106].

Once modifiable risk factors for dementia has been accurately recognized. Artificial intelligence (AI) may help bridge these gaps by enhancing our ability the integration of information about risk factors to model, forecast, and stratify populations, while identifying appropriate participants for clinical trials, as well as facilitate the identification of targets for preventative interventions. AI methods can be employed to evaluate the effectiveness of lifestyle and clinical interventions, for example, by modelling the effects of lifestyle modifications such as exercise and dietary modifications, as well as medical treatments, such as management of blood pressure, on dementia onset and progression [72].

In Alzheimer’s Disease, personalized treatment plans utilize AI to customize interventions according to individual patient profiles, by leveraging genomic, clinical, and lifestyle-related data to maximize therapeutic efficiency. These strategies encompass precision medicine to optimize pharmacological therapies and lifestyle modifications to particular patient profiles, enhancing therapeutic effectiveness while mitigating adverse outcomes [5]. Lifestyle-based strategies, encompassing exercise programs, cognitive stimulation, and dietary interventions, have demonstrated potential in decelerating Alzheimer’s Disease progression and enhancing cognitive performance. These measures can be personalized according to patient’s requirements, potentially directed by AI-enabled personalization to enhance treatment outcomes.Reddi Sree et al., [84]

Therefore, AI bridges the gap in risk-factor discovery to personalization research-based prevention, transforming diverse multimodal data into scalable, accurate strategies to reduce the incidence of dementia at both personal and population levels.

### AI-driven clinical trials

The unprecedented scope and intricacy of accessible data in the digital era offers a unique opportunity to advance our knowledge regarding Alzheimer’s disease and an enabling platform for the design of clinical trials and the development of novel drugs. Artificial intelligence (AI) approaches can facilitate the acceleration of drug discovery, advance insights into disease pathology, and improve the efficiency of clinical trials. AI can optimize multiple facets of the clinical trial process, including selection of patients, simulation modeling, and outcome forecasting. This approach may lower costs, accelerate timelines, and enhance the probability of successful outcomes Doherty et al., [30].

Artificial intelligence-enabled drug development and clinical trial design are central to efforts against Alzheimer’s disease. Machine learning (ML) and deep learning (DL) methods are employed to uncover novel therapeutic targets, forecast therapeutic efficacy, and enhance clinical trial planning. In clinical trials, AI can refine patient grouping, forecast treatment response, and decrease the likelihood of trial failures by selecting appropriate candidates and refining trial protocols. AI-driven personalized therapies integrate comprehensive omics, clinical, and lifestyle datasets to customize interventions and enhance therapeutic outcomes [30]. For example, in the AMARANTH AD clinical trial, an AI-guided predictive prognostic model (PPM) was employed for enhanced stratification of patients, integrating multimodal data to forecast disease progression with strong interpretability and it decreased sample size requirements by 30% while enhancing outcome evaluation and trial efficiency [96].

### Personalized treatment strategies

AI-driven personalized therapies incorporate comprehensive multi-omics, clinical, and behavioral data to tailor interventions and strengthen therapeutic outcomes. For instance, the Multi-Perspective Neural Network Ensemble Learning for Drug-Target Affinity (MNNEL-DTA) model utilizes deep learning to predict drug-target interactions by combining diverse multi-omics features, facilitating the repurposing of approved compounds for AD subtypes and optimizing therapeutic approaches to individual genomic profiles with enhanced performance as compared to traditional methods [94]. These advancements underscore AI’s pivotal role in transitioning from conventional uniform treatments to precision-based strategies that mitigate heterogeneity in AD progression and therapeutic response.

## Challenges and ethical considerations

Recent advances in machine learning and artificial intelligence have drawn a lot of interest in Alzheimer’s disease detection since they can identify the illness early. The development of AI (Artificial Intelligence) and ML (Machine Learning) for the diagnosis of AD (Alzheimer’s Disease) requires careful attention to ethical and regulatory issues. It is still unclear how AI-driven approaches to investigating the human brain will affect normative tools in research ethics and neuro-ethics and meet relevant standards of scientific validity given their transformative nature. Although new research suggests that Al may be useful in identifying early indicators of Alzheimer’s disease in speech, using data from people who are experiencing cognitive decline presents several ethical questions that should be talked about, taken into account, and resolved including:

- Privacy along with protection of personal data ((including the handling of personal content and medical information).
- Welfare (including distress, discrimination and reliability).
- Transparency (including the interpretability of language features and Al-based decision-making for developers and clinicians).
- Fairness (including bias and the distribution of benefits).
- Autonomy, i.e., consent of an individual as well as fairness (including consent, depersonalization and disclosure) [80].

### Even though AD has no known cure, early identification is preferred for a number of reasons

1. Preventive lifestyle modifications could be implemented, and early therapies could decrease the progression of the disease.
2. Access to programs that could ease the financial and psychological strain on patients and their families and aid in adjusting to the condition could be obtained.
3. Patients could be given the authority to act as self-determining agents and make decisions about their future Sanjaykumar et al., [86].

### Categories of AI-associated conflicts

The gap between the involvement of AI and ML in diagnosis and treatment for AD and the overall impact on society, especially in ethical aspect, can be divided into 4 broad categories:

#### Autonomy

In general, it refers to respect for a person’s freedom to self-govern, which implies freedom to choose according to one’s own judgement and moral principles without interference or manipulation. In AD, issues that could arise include According to the Declaration of Helsinki, consent needs to be ‘voluntary, competent, informed and comprehensive’, which are impaired in AD, thus increasing vulnerability of patient and altering the validity of informed consent. In any stage of the disease, the participants need to be made aware of the benefits and risks of the research prior to taking part in the study, understand what is being consented to, and how their data will be used. In case of deceased individuals, gaining the consent of their families can be challenging due to personal beliefs or limited contact. Patient autonomy, diagnostic ambiguity affect dissemination of research findings. Absence of treatment, inability of individuals in later sages to understand the diagnosis, and potential psychological reaction influence the transparency, which as of now is not evident in clinical procedures due to absence of obligation Petti et al., [80], Sanjaykumar, [86].

#### Privacy and data protection

Collection of data, analysing, storage and interpretation via aid of ML based NLP poses risk to the identity, legitimate information, and health-related sensitive data of patients with cognitive decline. Due to little control of researcher over the sort of data being extracted or required length of session, privacy concerns arise for AD patients. Furthermore, sharing and keeping voice data may increase the possibility of identification, particularly if the speaker is known to the listener Guan et al., [38].

#### Discrimination and stigmatization

Discrimination of those with early or pre-symptomatic AD can manifest in issues with access to social resources and opportunities. Due to the myths and misconceptions surrounding AD, stigmatisation can occur in both personal and professional contexts. Recognition of the illness as a distinct entity from the individual when insensitive psychometric tests are used to highlight the communicative impairments is challenging and such depersonalisation undermines autonomy. Public access to detect AD via speech would mean ability to test anyone within the microphone range, and gaining access to private clinical information, personal life, and further risk non-symptomatic individuals to develop psychiatric disorders. Historical injustices frequently give rise to algorithmic biases in the healthcare industry, which provide erroneous connections between protected class identification and disease outcome in the dataset. This is especially true when the underlying cause factors are found to span socioeconomic determinants of health Chen et al., [23].

#### Model adequacy and data bias

Psychological exams may contain integrated biases, such as the inadequacy of validation for varying educational attainment and the disparity in the calibre and quantity of published research. The repetitiveness of the method also poses a risk of habituation, compromising accuracy. The speech may be coherent, but it may describe events that are difficult to verify, and accurately determine if the speech is semantically valid. Current automatic emotion identification systems are unable to handle emotional variabilities; thus, constraints of emotion analysis should be made explicit. The multivariate character of the data and the differences in audio quality are two further possible biases associated with language and speech data. Cognitive bias on the part of researchers, or interpretation of information as already existing belief alters the quality of publications, giving rise of publication bias. Human bias is incorporated into the computer’s decision-making process with every human decision made during the construction of supervised machine learning models. Cultural background increases the possibility of misinterpreting communicative performance since it might influence language use and cognitive processes. Test accuracy is consistently greater for models trained on smaller datasets, but they typically perform poorly in real-world scenarios due to lack of data, and less sampling size. Data is the bottle neck of AI, making AI systems are only as good as the training data and underlying presumptions they are built on. Variability in the population and region it is extracted from, causes its approach to be unsuitable for varying scenarios. Models that are tuned for maximum accuracy may ignore fairness considerations, leading to persistently different performance across subgroups. Patterns from the dominating category of the data are likely to be reinforced when an algorithm is trained on biased data Norori et al. [75].

### Integration challenges in existing healthcare frameworks

It is challenging to integrate AI into existing healthcare frameworks due to concerns of experts regarding its impact on medicine and society at large.

#### Technical and performance limitations

A certain amount of scepticism is necessary because algorithms can overfit—perform poorly on other data after being optimised for a certain validation set. External or non-simulated environment testing for models is rarely done, which can produce inaccurate results, compromising the patient. Over time, a model’s predictive performance tends to decline, as patient demographics, medical procedures, and database definitions change, the reality may differ significantly from the dataset’s depiction, increasing the need to update algorithms frequently, which is a challenging task, especially due to a lack of data Guan et al. [38].

#### Data and infrastructure hurdles

Administrative, clinical, financial, patient-generated, and a wide range of research data are among the many different kinds of healthcare data, whose fragmented nature highlights the significance of interoperability of data for proper usage by AI devices. One of the main obstacles to the adoption of AI-assisted products is variable technical performance. Higher perceived dangers are a result of public scepticism in AI’s diagnostic capabilities and anxiety about AI performance [57]. Institutions must upgrade their EHRs to integrate AI, remove outdated equipment, install new data servers, and implement encryption to guarantee effective AI model performance, sufficient processing power such as a multicore, high-performance GPU or cloud-based servicesis required [48].

#### Trust, interpretabilty, and clinical impact

According to providers, AI-enabled products could result in employment losses. Physicians worry that clinical skills may be undermined by AI. Experts predict that medical gadgets with AI capabilities will transform the healthcare sector, impacting how doctors manage, diagnose, counsel, and triage their patients, along with the clinician’s intuition being hampered by cognitive overload. Patients worry that a machine’s decisions will not take into consideration their values or their personal care objectives. Cultural change and strategic planning would allow organizations to embrace AI solutions, which is a new concept that faces skepticism by experts. Society associated issues such as privacy concerns, stigmatization, mental stress, discrimination are issues discusses previously which are important during consideration of integration of [48].

The “reasoning” of machine learning algorithms, particularly multi-layered neural networks, differs from that of human reasoning. In essence, these “deep learning” algorithms are intricate systems of linked equations with several variables that do not match real-world notions. Therefore, there is no “reason” for a specific recommendation that is humanly understood. In order to assess if all relevant factors are taken into account, clinicians need to comprehend the rationale behind the recommendations, which is challenging with such “black box” AI systems. Because of intricate internal architectures, the “black-box” problem in AI restricts interpretability and makes it challenging for developers to forecast or justify model decisions [57]. Limitations on sample size, improper statistical significance, and self-serving biases like “data shopping” are important factors to take into account when understanding loopholes of AI use in healthcare [103].

### Ethical roadmap for AI in ad

Features involved in the standardised acronym SHIFT (Sustainability, Human Centredness, Inclusiveness, Fairness, Transparency) encompasses aspects like privacy-preserving federated learning Model, ethical and regulatory safeguards, frameworks addressing bias mitigation (balanced training cohorts), informed-consent for data use, and transparent disclosure of results to ensure fairness and safety. Continuous post-deployment monitoring and adequate support would be crucial for avoiding depersonalization, discrimination or stigmatization. Encryption of data, licencing of speech analyser and monitoring its use is important to preserve privacy. Algorithmovigilance and frequent updating of algorithms is essential since AI, especially black-box AI, can prove unreliable in cases involving rigid datasets, lack of data and exposure, and updates in patient demographics, and medical procedures overtime. It is indispensable to upgrade EHRs in clinical institutions to integrate data, remove outdated equipment, install new data servers, and implement encryption to guarantee effective AI model performance. Thus, integration of AI in clinical practices poses various challenges which can be overcome using such counter-active measures.

## Recent findings related to application of AL/ML in Alzheimer’s disease

### Transformer-based models for multimodal dementia data

Transformers, which make extensive use of the attention mechanism that supports many NLP models, were introduced by Vaswani et al., Models using features defined by experts can only select from a small number of predefined features, while transformer-based language models are able to extract more intricate and context-related features with the help of data availability and fine tuning. Multilingual models are among the most popular categories of transformer-based language models. By applying the knowledge of AD prediction from another language where a large dataset is available, the issue of not having access to a large dataset in one language can be resolved with the appropriate usage of multilingual models. The necessity of having specialists in the target language describe linguistic aspects is also addressed by such transfer. BERT (Bidirectional Encoder Representations from Transformers) is a recently found attention-based model designed to significantly improve the efficiency and accuracy of Natural Language Processing (NLP) tasks, being pre-trained on an a substantial corpus and has a bidirectional transformer network, alongside getting fine-tuned to solve a variety of linguistic tasks. The task-specific BERT architecture, to put it briefly, uses sequential tokens to represent input text. The tokens and segmentation, along with position embeddings create a combination which is utilized to form the input representation. The proposal of BERT model put forth by Devlin et al., was the turning point in transformer-based language models. Cross-lingual language model (XLM), an improved version of BERT for multilingual language comprehension tasks that makes use of both the masked language model (MLM) and the translated language model (TLM) was presented by Conneau et al., The independent prediction of masked tokens and the discrepancy between the train and test phases limit BERT, and were corrected issue by introduction of an extended large network (XLNet) model that was based on the Permutation Language Model, a language model. A practical answer to the issue of a lack of a sizable dataset in numerous languages is the application of multilingual models. Training a multilingual model in a source language (where such vast datasets are available) and using it to make inference in the target language can be a useful approach since there is a limited collection of text data from Alzheimer’s patients in many languages. Models based on linguistic characteristics outperform predictive ones based on non-linguistic variables (e.g., age, gender, APOE 4 alleles, neuropsychological test results, etc.). The use of pre-trained deep transformer-based language models allows for the extraction of more sophisticated features from the data while lowering the requirement for expert-defined language features. In AD risk assessment, these models with a basic logistic regression classifier can perform well, and the outcomes typically surpass those of the current approaches. Additionally, the suggested approach eliminates the need for manually created features for classifier training. The task for gathering voice data from the patients is the Cookie-Theft visual description test. Previously used to diagnose aphasia, the test is currently used by speech-language pathologists frequently utilise the test to evaluate individuals with diseases such schizophrenia, aphasia, AD, right hemisphere lesions, and others for aberrant language production (Roshanzamir et al., [ 33, 64, 85]).

Future directions include the enhancement of multilingual AD prediction by utilising cross-lingual transfer learning in conjunction with pre-trained multilingual transformer-based language models, addressing the issue of inefficiency of typical techniques for interpreting neural networks because of the high number of model parameters [85].

Despite the promising performance reported across these studies, substantial methodological variation is evident. Most models were trained and evaluated on limited or publicly available datasets, particularly ADNI, restricting population diversity. Several investigations relied primarily on internal cross-validation without independent external cohorts, increasing the risk of overestimated accuracy. Differences in sample size, follow-up duration, outcome definitions, and class imbalance further limit direct comparison. Although multimodal approaches generally achieved superior performance, their complexity and data requirements reduce feasibility in routine clinical settings. These findings emphasize the need for standardized evaluation protocols and large-scale prospective validation [26, 104].

### Generative AI

#### Application in pet scans

A generator that analyses the possible distribution of real data to produce new data samples along with a discriminator (binary classifier) which recognises the most accurate distinction between generated and real samples make up the potent class of generative models known as Generative Adversial Networks (GANs). The two components learn and function simultaneously, while having the potential to adopt to the architecture of widely used deep neural networks [100].

When diagnosing brain diseases like Alzheimer’s disease, combining data from multiple modalities often results in better performance than when utilising only one modality.

However, because it is hard to get complete data that covers all modality data in clinical practice, training a multi-modality model remains problematic [61].

Understanding Alzheimer’s disease requires examining the spatiotemporal patterns of amyloid buildup in the brain across time. Since it makes it possible to see and measure the aberrant amyloid beta (Aβ) load in the living brain, Positron Emission Tomography (PET) imaging is essential for monitoring the course of the disease and assessing the effectiveness of anti-amyloid treatments. Generative artificial intelligence is capable of producing lifelike synthetic visuals and learning intricate data distributions, along with production of rich representations for statistical prediction, progression modelling, and simulating evolution in artificial patients. In order to comprehend complex data distributions and produce realistic synthetic data, generative artificial intelligence (AI) uses sophisticated models such as variational autoencoders (VAEs), diffusion models, and GANs. By artificially increasing the pool of images available for training, these models have demonstrated their ability to improve algorithm performances and lessen the problem of data scarcity. By creating artificial images and mimicking the changes of a fictitious patient over time, progression models go beyond image production. MRIs have become indispensable and are generally followed by brain parcellation, mapping of brain segmentation to the PET image, and finally a group comparison of normalized PET signals within each region. Recently, some researchers have proposed deep learning-based automatic quantification techniques that only use amyloid PET images to get over these restrictions.

These networks are particularly trained to calculate a few regional SUVR averages, assess amyloid positive, and estimate the cortical-to-cerebellum standardised uptake value ratio (SUVR) average. But this consequently results in the loss of the data’s rich spatial information. GANs create realistic-looking features, including imputing missing PET images, by learning the sample distribution from actual data This offers a potent method of directly modelling and modifying images. Encoding PET pictures in an accessible location that may be utilised for many analysis later is an alternate strategy that is gaining attention in present times. This representation offers a strong modelling framework for analysis and simulation when combined with a generative process.

StyleGAN-based architectures offer use in medical imaging, MRI evaluation, and the creation of remarkably realistic and varied images. In comparison to earlier generative models, they also have an intermediate latent space that is less entangled than the input space, demonstrating improved control and interpretability on picture synthesis.

A recent study proposed to leverage an extension to StyleGAN, a 3D GAN model called 3D StyleGAN for PET image synthesis and representation. By using a linear regression model to forecast SUVR and assess the performance degradation when projecting the latent vector in even lower-dimensional spaces provided by Principal Component Analysis, it provides a way to measure the latent space information richness. Amyloid dynamics on a five-dimensional PCA subspace of the latent space were also modelled using a non-parametric ODE model based on Gaussian Process (GP) regression. It is the first study to study amyloid progression in PET, while prior studies employed it to model neuroanatomical changes and progression only from MRI scans Bossa et al., [18].

#### Application in speech marker biosynthesis

One of the first indicators of cognitive decline is language impairment, which can also be used to distinguish AD patients from healthy people. This makes it a low-cost, non-invasive method of conducting extensive AD screening. However, creating reliable data-driven speech-based AD diagnostic algorithms is severely hampered by the scarcity of AD speech samples. A recent study suggests Reverse-Speech-Finder (RSF), a novel domain-specific speech-based AD detection method that uses large language models (LLMs) for fine-grained speech analysis, including the identification of novel speech markers and the creation of data based on those markers, in order to address these issues. To identify important speech signals that predict AD, RSF offers a revolutionary reverse engineering approach to AD detection. It takes advantage of the finding the most likely speech markers (MPMs), which are speech markers most likely to predict AD, must also have the highest probability of activating the most probable neurones (MPNs), which are neurones in the neural network with the highest probability of predicting AD. It employs a speech token representation at the input layer, which enables the most likely speech tokens (MPTs) of AD to be found by backtracking from MPNs. Last but not least, it creates a creative backtracking technique to trace backwards from the MPNs to the input layer, locating the MPTs and matching MPMs and cleverly revealing new speech markers for AD identification. Results from experiments show that RSF is better than more conventional techniques like SHAP and Integrated Gradients. RSF greatly improves the accuracy and robustness of AD diagnostic models while also reducing the constraints of real data scarcity by producing speech data that contains novel indicators. These results highlight RSF’s promise as a game-changing technique for speech-based AD detection, providing fresh perspectives on language impairments associated with AD and opening the door for more potent non-invasive diagnosis and prevention [59].

Another study seeks to produce text embedding, a vector representation of the speech-to-text transcribed text, that captures the semantic meaning of the input by utilising the extensive semantic knowledge included in the GPT-3 model. It has been effective in differentiating AD patients from healthy controls and determining the subject’s cognitive test score based only on speech data. It also demonstrates that text embedding performs competitively with existing fine-tuned models and significantly beats the traditional acoustic feature-based approach. Three regression models support vector regressor (SVR), ridge regression (Ridge), and random forest regressor (RFR) are used to estimate a subject’s MMSE score for the AD severity prediction regression analysis, which is based on both the auditory characteristics and GPT-3 embeddings [2].

### Graphical neural networks for patient-level disease mapping

Traditional deep learning architectures, including recurrent neural networks (RNNs), convolutional neural networks (CNNs), and multilayer perceptrons (MLP), have been used to examine conditions associated with cognitive decline. The developments in neuroimaging modalities such as PET, MRI, diffusion tensor imaging (DTI), and functional MRI (fMRI), have increased the efficiency of monitoring, diagnosis, and prognosis remarkably. The network-like characteristics of brain data are difficult for traditional DL techniques like CNNs to capture since AD causes anatomical and functional changes in brain connectivity. To overcome these difficulties, graph-based techniques have been developed, which describe local interactions, subnetworks, and brain connectivity in a way that is more biologically realistic Pasquini et al., [78].

Despite being widely used in computational-aided diagnostic systems, CNNs face considerable difficulties when processing multimodal neuroimaging data. These drawbacks include the need for consistent input dimensions across channels, restricted interpretability when integrating multimodal data, and their incapacity to take inter-subject correlations into account. Technological developments in GNNs provide promise for improving AD diagnosis through the analysis of multimodal neuroimaging data. GNNs are an advanced subset of artificial neural networks made especially to process data with graph structures. A graph, represented formally as G = (V, E), is a collection of nodes V and edges E that reflect pairwise relationships between items. Edges show the connections between nodes, which stand in for entities. Early studies that applied neural networks to directed acyclic graphs served as inspiration for the fundamental ideas behind GNNs. Brain networks created from sMRI or PET images can be employed in a population graph framework that combines the imaging properties of these networks with phenotypic data. A method for combining the multi-modal data at the level of adjacency matrices and node vectors is suggested, and a multi-modal GNN framework is described, with each modality having its own branch of GNN. To create a final forecast, late fusion is used to integrate the initial choices made in each branch. The trend in AD diagnosis is multi-source and multi-modal, as multi-modality data is made available [111].

The capacity of GNNs to operate on non-Euclidean data is one of its distinguishing features. On the other hand, conventional ML and DL techniques are less useful for graph-structured data since they are mainly made for Euclidean data formats, such pictures or sequential text. By using local message consolidation and transmission across edges, GNNs overcome this restriction and allow nodes to methodically collect data from their neighbours and improve their representations. GNN variants based on CNNs and graph embedding are suggested as a way to collectively gather information from graph structure. As a result, they can simulate input and/or output made up of components and their interdependencies. Although DeepWalk and other early embedding techniques used random walks, they were limited in their capacity to share parameters and generalise to fresh or dynamic graphs. In order to portray elements and their dependencies collectively, GNNs combine data from the graph structure. They can be sorted into four categories: spatial-temporal GNNs, convolutional GNNs, recurrent GNNs, and graph autoencoders. The core mechanism can be summarized as graph construction by data points stimulation, message passage and aggregation of immediate neighbours and nodes information in nodes and use of final learned node representation for prediction. Graphs may be of a few types including structural (explicit structure using molecules, knowledge graphs), and non-structural (implicit; graph is built from the task). They may be directed or undirected, static/ dynamic (topology or features change with times), homogenous/heterogenous (based on nodes and edges types). The GNN comprises of propagation module for capturing feature and topographical data, sample module to conduct propagation on large graphs, and pooling module to extract information from nodes to obtain high-level subgraph or graph representation integrated together. A few models based theoretically on graph signal processing which define convolution in spectral domain include spectral network, ChebNet, Graph Convolutional Network, Adaptive Graph Convolution Network, Dual Graph Convolutional Network, and Graph Wavelet Neural network.

Graph neural networks have developed into strong and useful instruments for machine learning tasks in the graph domain in recent years. This development is due to improvements in training techniques, expressive capacity, and model adaptability [112].

GNNs have been used in the biomedical field for tasks like disease classification, drug discovery, protein function prediction, and personalised medicine. GNN models, such as the variationally regularised encoder-decoder GNN, which was created especially for electronic health records, have shown promise in revealing biological insights, revealing hidden patterns, and advancing ADRD prediction. But even with some sophisticated models like GAT, GCT, and VGNN, the GNN architecture is still a black-box model, and its lack of interpretability is detrimental to users and society as a whole, particularly in crucial applications where judgements must be clarified or understood [42].

## Future innovations and direction

Future directions include enhancement of multilingual AD prediction by utilising cross-lingual transfer learning in conjunction with pre-trained multilingual transformer-based language models, addressing the issue of inefficiency of typical techniques for interpreting neural networks because of the high number of model parameters [85].

In order to increase the accuracy of detection methods, future research will concentrate on removing redundant and unnecessary characteristics from existing feature sets as well as on extracting and analysing new features that are more likely to help with Alzheimer’s disease diagnosis. Alogrithms can be trained to differentiate between healthy persons and those with Alzheimer’s by including variables like MMSE and education [52]. Developing AI methods in focus include task analysis, the integration of multimodal data, and deep neural networks [91].

### AI architecture and multimodal data integration

Due to symptom overlap across aetiologies, differentiating dementia diagnosis is difficult in neurology, but it is essential for developing early, individualised therapy plans [107].

Integrated multimodal data pipelines combine neuro‑imaging (MRI, PET, CT), genetics/proteomics, electronic health records, and speech‑language biomarkers into a single learning framework. Fusion is performed with deep‑learning models (CNNs for images, transformer‑based encoders for text/audio) that learn shared latent representations, improving sensitivity to subtle disease signatures [49]

In semi-supervised techniques like active learning, a model is trained on a labelled data set, which is then used to identify the most uncertain or instructive examples from the unlabelled data set. Active learning could be used for a number of dementia preventive tasks, like finding pertinent risk factors or biomarkers or selecting specific samples for clinical studies. To find and evaluate new possible drug-repurposing candidates for dementia prevention, further techniques like deep learning and network-based approaches will be essential. Clinical translation of risk ratings and machine learning algorithms could be achieved with ease by utilising data that is already regularly gathered in clinical settings. However, combining a variety of data modalities, such as genetic, multiomic, and advanced neuroimaging features, could increase sensitivity and specificity, but at the expense of clinical transferability, and would necessitate a higher level of resources, which many populations do not have. Even if applying an ML technique could improve the analysis of multimodal, high-dimensional data, this often results in increasingly complex models becoming uninterpretable [72].

### Clinical applications: early detections and prognostic modelling

Early‑stage detection AI involves enhanced imaging extracts quantitative biomarkers (e.g., cortical thickness, amyloid‑PET uptake) that precede clinical symptoms. Speech‑analysis models (acoustic‑prosodic and lexical features) detect language impairments months before cognitive tests become abnormal. Joint image‑speech models achieve higher accuracy than any single modality, enabling pre‑clinical screening. Personalized therapeutic decision‑making Machine‑learning classifiers predict individual drug‑response profiles by linking multimodal phenotypes to pharmacodynamic data from virtual‑screening pipelines. Continuous monitoring of multimodal biomarkers (imaging changes, speech patterns, medication adherence) allows dynamic adjustment of treatment plans. Prognostic modelling of disease trajectory Recurrent or temporal‑attention networks ingest longitudinal multimodal records to forecast conversion from mild cognitive impairment (MCI) to AD and to estimate future cognitive scores (e.g., MMSE). Risk‑stratification outputs support clinicians in planning long‑term care and trial enrolment. (Kale M. et al., n.d.)

Model predictions are validated with gold-standard biomarkers (amyloid PET, tau, FDG) and postmortem pathology, demonstrating close alignment with underlying disease mechanisms and reliability for clinical translation. AI guidance improves the accuracy of radiologist interpretation of MRI scans in dementia cases. The innovation includes transformer architectures able to integrate heterogeneous input (history, imaging, functional tests) and deliver reliable results even when substantial data are missing. Probability scores for stage and severity (linked to CDR, MMSE) allow stratified care planning without requiring explicit staging, bridging gaps in clinical workflow. The model can be deployed in diverse healthcare environments (primary care, memory clinics, tertiary centers), supporting scalable, accessible diagnosis in settings with limited specialist access. The system increases objectivity and reduces variability in clinical assessment, helping standardize dementia care and inform trial recruitment, especially important given shortages of trained neurologists. Innovations support patient screening for clinical trials via robust differential diagnosis, not just early detection, broadening AI’s impact from diagnostics to research and therapy planning [107].

### Ethical, privacy and regulatory frameworks

Privacy‑preserving federated learning Model training is distributed across participating hospitals; only encrypted weight updates are shared, keeping raw patient data on‑site. Reported federated models reach AUC ≈ 0.94 while satisfying HIPAA‑style data protection. Ethical and regulatory safeguards Frameworks address bias mitigation (balanced training cohorts), informed consent for data use, and continuous post‑deployment monitoring to ensure fairness and safety [49].

The idea of “algorithmovigilance” stresses the need to continuously assess AI algorithms in order to reduce bias that might arise due to sample size, historical bias, representation bias, sponsorship bias, and self-serving prejudice in the development process. For the responsible application of AI in healthcare, standardised acronyms such as “SHIFT” (Sustainability, Human Centredness, Inclusiveness, Fairness, Transparency) might aid in reaching a framework on important AI concerns and patient and community protection measures [103].

### Role of explainable AI (XAI) in clinical setting

Recent years have seen an exceptional expansion in computational capability, which has made it possible to create Artificial Intelligence (AI) models for medical applications with significant results. However, because AI models are typically blackboxed, many AI-powered Computer-Aided Diagnosis (CAD) techniques have not been widely accepted or adopted in the medical field. Therefore, the predictions made by these AI models (decisions, recommendations, and guidance) need to be comprehensible and interpretable to encourage medical professionals to use them for gaining new knowledge. Justifying the reliability of these models’ predictions is the goal of the nascent discipline of explainable AI (XAI). Explainability for a model decreases with an increase in its complexity and performance quality. For an AI model, XAI would aid it to identify and rectify the errors if it generates adverse results, with an accurate explanation of its working and influential factors considered for prediction [99].

### XAI techniques & application associated with alzheimers disease

The XAI techniques can be roughly divided into four groups according to the following criteria: (i) explanation scope, (ii) implementation stages, (iii) model applicability, and (iv) explanation forms. There are numerous studies on AI-based AD detections that use XAI, including implementation of multi-layered multi-model system for an correct and interpretable AD diagnosis, proposal of strong framework for classification among Healthy Control (HC), Mild Cognitive Impairment (MCI), and AD and explain the predictions with methods associated to XAI, use of trustworthy multi-class classification model supported by XAI methods to interpret the predictions correctly, proposal of a computer approach called Systems Metabolomics utilising Interpretable Learning and Evolution (SMILE), discovering and identifying the most instructive metabolites to comprehend and diagnose the onset and progression of disease, examination of tau, beta-amyloid, and neurodegenerative biomarkers for the categorisation of AD, comparing the prediction and interpretability capabilities of the top three models from “The Alzheimer’s Disease Prediction of Longitudinal Evolution” (TADPOLE) challenge using a common XAI framework. To conclude the causes and markers of Alzheimer’s disease, the high performance of the model (XGBoost) was used in conjunction with explainable machine learning techniques that could analyse the relationships between the different features [16].

### Challenges in application of AI

Because there is disagreement about what constitutes interpretability and the range of available methodologies, it can be challenging to develop formal criteria and an enhanced systematic evaluation of various approaches. Furthermore, when assessing explanations of a black-box model, there is no ground truth, in contrast to standard performance measurements. Despite being more understandable, it is considered as simplified as opposed to the complex and apparently reliable nature of other DL models like CNNs, meaning there is a trade-off among explainability and prediction accuracy. Some clinicians believe that emphasis on the interpretability of models might restrain innovation before there is clinical implementation [73].

Transparency and explanations might not be synonymous to the result of grasping the fundamental causal links in biology, hence it is treated with caution in the aspect of knowledge discovery and enhancement. Explanationsex rely on the input data and users request, and inadequate data or vague query might hamper the result quality [74]. There is a significant amount of practical competence that is specific to patient and expert encounters in clinical practice, where reproducible experimentation is at least somewhat feasible, and there is a dearth of generalisable information for many real-world problem-solving scenarios. In order to help data scientists and engineers incorporate interpretability in artificial intelligence for healthcare in a more thorough, validated, consistent, and comparable manner throughout the entire methodology, layout, and algorithmic development process, more reliable metrics, standards, and approaches are required. The metrics based on intuitions mathematically measure the similarity between explanations and instances as well as the agreement between the explainer and the black-box model. Qualitative intuitions are beliefs about the cognitive form, complexity, and structure of the explanation, while quantitative intuitions are easier to formally measure and include ideas like identity (similarity between situations and outcomes), stability (comparability between instances from the same category and outcomes), or separability (distinct nature between different instances) [35, 99].

## Future developments in xai in the healthcare sector

Undoubtedly, knowledge from experts would aid in the development of stronger AI systems with improved clinical interpretability. Research on different approaches of incorporating clinicians’ knowledge into AI models will therefore be highly beneficial (RQ3 and RQ4), through practices similar to human-in-the-loop evaluation for such systems.

A model’s diagnostic accuracy and interpretability can be enhanced by combining various forms of multimodal data, including genetic information, family histories, medical pictures, and electronic health records, to help define, characterise, and include clinical context. Additionally, employing data gathered from many sources helps improve AI models and assist with domain variability. In order to enhance existing explainability techniques and create and apply new and more efficient ones, technical designers must learn and be imbued with the ability to detect errors in models related to a specific field of application. Users’ needs must also be taken into account more explicitly. For precise explanations to meet the demands of consumers to be informative and intelligible to them, it may be better to develop various sorts of explainability, depending on the potential end users of the descriptions, whether they be patients, doctors, or technical designers. Interaction between the system and the individual user may also be necessary to achieve this user knowledge to get further responses to various queries [35].

### Advancing global collaborations for accessible AI solutions

Partnerships place a strong emphasis on open governance, stringent bias mitigation, and community-engaged informed consent procedures to make sure AI solutions reduce rather than exacerbate health disparities, particularly among diverse populations around the world. By focusing on include under-represented communities that are frequently left out of Alzheimer’s research, global genetic and clinical cohorts are being created that will uncover new biomarkers and risk stratifications that will inform individualised preventative efforts across the globe. For example, in order to ensure equal access and impact, the Africa Task Force on Brain Health brings together regional specialists to integrate cutting-edge AI solutions with local health concerns. This involves customising prevention and care technologies for settings with limited resources [9, 28].

Open, secure research platforms have enabled the researchers to connect and work efficiently. AD Workbench is a secure, cloud-based platform that allows researchers worldwide to find, access, and share multimodal Alzheimer’s data, eliminating data silos and encouraging collaborative analyses which accelerate novel prevention and treatment discovery. With more than 6000 users from 115 countries, including many from low- and middle-income countries, AD Workbench democratises access to rich datasets while maintaining controlled security and interoperability. Alzheimer’s research is shifting from late-stage diagnosis to predictive and preventive science using AI-powered analytics thanks to public-private partnerships, opening up possibilities for managing brain health throughout one’s life Au et al., [9].

Global clinical trial networks expand geographic access to trials and expedite regulatory assessment and uptake by using AI-powered recruitment and monitoring to speed up the testing of preventive therapies[27].

Supported by philanthropists like Bill Gates, the Alzheimer’s Insights AI Prize provides $1 million to develop autonomous AI systems that maximise Alzheimer’s prevention, spurring innovation and worldwide engagement to accelerate solutions. Such collaboration encourage participation and progress in research alongside increasing awareness [69].

### Potential role of AI in diagnosis and prevention of AD in future

Introduction of AI in the field of medical science broadens the horizons significantly in all relevant aspects. With multimodal data pipeline integration being done presently, neuroimaging, genetics, EHRs, and speech language biomarkers can be integrated into a sole structure as multimodal data, leading to better detection, relevant therapy and efficient monitoring, alongside multi-omics research which promises early detection through computational modals. Amyloid deposition quantification with aid from AI and ML, especially deep learning modals proves to yield more accurate and cost-effective methods relative to traditional ones, while making population‑wide amyloid screening financially feasible and fast enough for preventive programs. Speech-language detection serves as a critical base for early detection of AD, which could potentially be established as a low-cost, non-invasive technique for AD screening given an adequate amount of speech samples. Age and APOE ε4 alleles are frequently used as a non-linguistic variable in deep transformer-based language models designed for AD risk assessment, and as a multimodal feature in hybrid classification models like Convolutional Neural Network-Support Vector Machine (CNN-SVM) frameworks, for use of information about these modifiable factors to model, forecast, and stratify populations. GNNs can integrate network-like characteristics of brain data from PET, MRI, DTI, and fMRI, amplifying the efficiency of monitoring, diagnosis, and prognosis remarkably while possibly enhancing ADRD prediction, uncovering hidden patterns, and uncovering biological insights. The issue of data scarcity, the bottleneck of AI, can be resolved using Multilingual Transformer-based modals. Involvement of AI accelerates drug discovery, enables personalized treatment strategies to customize interventions, and supports cognitive rehabilitation—by leveraging clinical, genomic, and lifestyle-related data to maximize therapeutic efficiency. A variety of dementia prevention tasks including the selection of particular samples for clinical research could benefit from the application of semi-supervised approaches such as active learning. Finding and evaluating novel potential drug-repurposing options for dementia prevention may need the use of DL and network-based approaches. Regularly collected data in clinical settings could facilitate the clinical translation of risk evaluations and machine learning algorithms. Privacy-preserving federated learning Model training is distributed across participating hospitals where raw patient data is kept on-site only. Regulatory and ethical protections Frameworks cover informed permission for data usage, bias prevention (balanced training cohorts), and ongoing post-deployment monitoring to guarantee safety and equity remain indispensable. Nevertheless, systematic bias monitoring, continual post‑deployment auditing, and transparent XAI explanations remain essential to ensure equitable, trustworthy AI deployment, a prerequisite for widescale preventive adoption, which is expected to occur in near future with the developments in the field of AI and ML and their integration into that of medicine.

## Conclusion

A paradigm shift in the early diagnosis, prevention, and detection of Alzheimer’s disease is represented by artificial intelligence and machine learning. AI models are able to detect subtle, preclinical signs of cognitive decline and make very accurate predictions about the course of diseases by combining multimodal data, such as genetics, neuroimaging, and electronic health records. These tools help customised preventative programs by identifying changeable risk factors and lifestyle changes, in addition to speeding up drug discovery and clinical trial design. However, ethical issues, including data bias, privacy, and model transparency must be addressed for clinical translation to be successful. Explainable, fair, and internationally cooperative AI systems that democratise access to preventive technologies and allow for precise brain health management across a range of demographics are the key to the future of Alzheimer’s care.

## Data Availability

No datasets were generated or analysed during the current study.
